# Cervical lymph node TB: diagnostic yield and patient profile

**DOI:** 10.5588/ijtldopen.25.0453

**Published:** 2026-02-11

**Authors:** F.R. Luba, P. Ghosh, S. Anwar, B.P. Dey, B. Bhowmick, S.M. Arafat, A.A. Saleh, M.S. Shomik, S. Maruf, S.K. Sagar, M. Ashaduzzaman, A. Ceruti, M. Siegel, C. Arpa, J.B. Okuni, S. Schneitler, D. Mondal, A. Abd El Wahed

**Affiliations:** 1 Nutrition Research Division (NRD), International Centre for Diarrhoeal Disease Research, Bangladesh (icddr,b), Dhaka, Bangladesh;; 2 Institute of Animal Hygiene and Veterinary Public Health, Leipzig University, Leipzig, Germany;; 3 Department of Empirical Health Economics, Technische Universität Berlin, Berlin, Germany;; 4 Bangladesh Medical University (BMU), Dhaka, Bangladesh;; 5 Chair of General Economics, Health Economics and Econometrics, University of Greifswald, Greifswald, Germany;; 6 Heidelberg Institute of Global Health, Heidelberg University, Heidelberg, Germany;; 7 College of Veterinary Medicine, Animal Resources and Biosecurity, Makerere University, Kampala, Uganda;; 8 Institute of Medical Microbiology and Hygiene, University Clinic Saarland, Homburg, Germany;; 9 Institute for Medical Microbiology, Immunology, and Hygiene, University Hospital Cologne and Faculty of Medicine, University of Cologne, Cologne, Germany.

**Keywords:** tuberculosis, Bangladesh, extra-pulmonary TB, diagnosis, cytopathology

## Abstract

**BACKGROUND:**

Cervical lymph node TB (CL-TB) is the most prevalent form of extra-pulmonary TB, yet it remains underdiagnosed in endemic settings due to non-specific symptoms and inconsistent diagnostic pathways. We aimed to identify socio-demographic and clinical predictors of CL-TB in patients attending a tertiary hospital in Bangladesh and evaluate the diagnostic yield of available tests.

**METHODS:**

We conducted this cross-sectional study at Bangladesh Medical University. We screened 3,619 cervical lymphadenitis patients and enrolled 104 with presumptive CL-TB. Lymph node (LN) aspirates were tested using microscopy, cytopathology, GeneXpert, polymerase chain reaction (PCR), and culture. Data were analysed using STATA 15.

**RESULTS:**

Among 104 participants, 52 were confirmed to have CL-TB by either microscopy, cytopathology, GeneXpert, PCR, or culture. Younger age (18–30 years) and purulent aspirate appearance were independently associated with CL-TB. Cytopathology showed the highest diagnostic yield (82.7%), followed by GeneXpert (71.2%) and PCR (67.3%), while microscopy and culture detected <7% of CL-TB cases.

**CONCLUSION:**

Combinations of cytopathology with GeneXpert or PCR yielded near-perfect CL-TB detection. This was the first study in Bangladesh to utilise LN aspirates directly for PCR alongside cytopathology, GeneXpert, microscopy, and culture, demonstrating a feasible diagnostic approach in high-burden settings like Bangladesh and offering improved detection and reduced delay.

TB is caused by *Mycobacterium tuberculosis* (*Mtb*), which is primarily transmitted between humans via airborne droplets and commonly affects the lungs. Additionally, the bacterium can infect other organs such as lymph nodes (LNs), kidneys, brain, and skin, a condition known as extra-pulmonary TB (EPTB).^1^ TB has overtaken other diseases to become the leading cause of death from a single infectious agent, causing 1.25 million deaths in 2023. An estimated 10.8 million new cases occur annually, of which approximately 15%–20% are classified as EPTB.^2^ Of these EPTB cases, the LNs are predominantly affected (in 60%–90%), particularly the cervical LNs.^3^ The involvement of cervical LNs can result from either direct extension of TB infection or its dissemination via bloodstream (haematogenous spread). Initially, the affected LNs are painless, firm, and discrete, which turn into fluctuating, matted masses. Eventually, they form abscesses with skin breakdown and chronic sinus formation, which gradually heal with scarring.^4^ A history of contact with TB patients with these clinical findings can lead to a provisional diagnosis of cervical lymphadenopathy due to TB (CL-TB). The presence of low-grade evening fever and unexplained weight loss, which are the classical signs of TB, may also be seen.^4^

A definitive diagnosis can be established through fine-needle aspiration cytology (FNAC), including microscopic examination of smears to identify acid-fast bacilli (AFB), rapid molecular testing with Xpert MTB/RIF to detect *Mtb* DNA and assess rifampicin resistance, highly sensitive Ultra culture methods to grow *Mtb*, and/or by biopsy for histopathological analysis.^4^ Despite their utility, these diagnostic tools are not without limitations. While microscopy offers advantages of low cost and simplicity, its reliance on high bacterial load (exceeding 10^4^ cells/mL) and poor sensitivity^5^ have led the World Health Organization to favour molecular tests like GeneXpert and polymerase chain reaction (PCR) for TB diagnosis. Despite the faster and more sensitive results offered by GeneXpert and PCR, their implementation is costly, demanding specialised laboratory and trained personnel.^6^ While culture is the gold standard, the slow proliferation of most pathogenic mycobacteria results in a substantial diagnostic delay. FNAC and histopathology are moderately sensitive and specific and require expertise, which may not be readily available in all district-level hospitals in Bangladesh.^4,7^ The existing literature indicates that DOT (Directly Observed Treatment) centres are not the primary health care entry point for EPTB patients.^8^ The absence of a comprehensive EPTB diagnostic service forces patients to engage with multiple providers for different investigations, a system that ultimately leads to diagnostic delays.^9^

In this nested study, we aimed to explore the detection rates of available diagnostic tests in Bangladesh and shed light on the socio-demographic and clinical aspects associated with CL-TB in patients attending a tertiary health care centre in Bangladesh.

## METHODS

**Figure 1.**
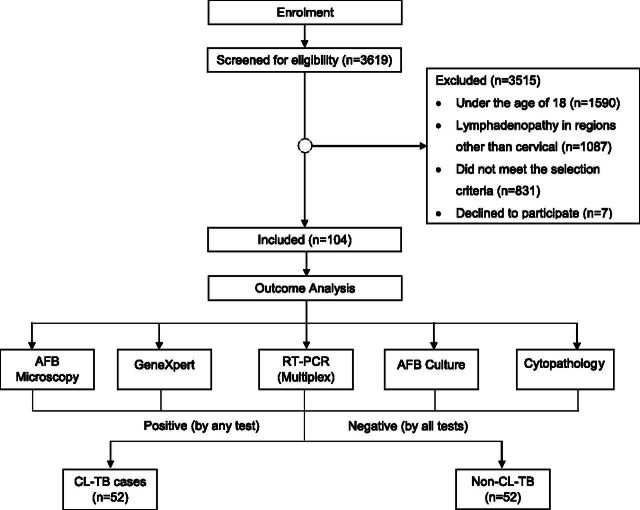
Flow diagram for the study, demonstrating the enrolment procedure and the division of the participants into two groups: cervical lymph node-TB (CL-TB) cases and non-TB participants with cervical lymphadenitis.

This cross-sectional study was nested within the ‘Diagnostic Algorithm for Peripheral Lymph Node Tuberculosis Using Portable Station/Mobile-TB-Lab’ study, a multi-country diagnostic evaluation spanning Germany, Uganda, and Bangladesh. In Bangladesh, the study site was Bangladesh Medical University (BMU). The study population was selected from the patients with lymphadenitis (LN swelling) arriving at the BMU Internal Medicine Outpatient Department (OPD), presenting with one or more enlarged, palpable cervical LNs, and either chronic low-grade fever (>2 weeks), or a history of contact with TB patients, unexplained weight loss, or cough. Patients with known diagnoses of cancer or infections other than TB were excluded from the study. We screened 3,619 patients from January 2024 to August 2024 and enrolled 104 patients (Figure 1).

### Study procedure

Following informed consent, we collected demographic and clinical data from each patient using a semi-structured questionnaire. The study physician performed a routine physical examination, maintaining the privacy of the patients. Fine-needle aspiration (FNA) from the affected LNs was then performed, adhering to standard aseptic techniques for microscopic examination for AFB and cytological evaluation. The remaining samples were utilised for AFB culture, real-time multiplex PCR (RT-PCR [Multiplex]), and GeneXpert (Cepheid, USA) at the Department of Microbiology & Immunology, BMU. We described the methodology for culture, R-T PCR (Multiplex), and GeneXpert in the Supplementary Data available on Figshare (https://doi.org/10.6084/m9.figshare.30302923). Following the dispatch of diagnostic findings, we informed patients and assisted them in registering at the DOT centre to ensure TB treatment. The diagnostic findings were used to stratify participants into CL-TB and non-CL-TB groups (52 in each group) based on the following operational definitions.

### CL-TB cases

CL-TB cases included participants who were presumed by clinicians as cases of TB lymphadenitis (TBLN) and tested positive through either AFB microscopy and/or cytopathology and/or culture and/or RT-PCR (Multiplex) and/or GeneXpert.

### Non-CL-TB

Non-CL-TB cases included presumptive CL-TB participants with negative diagnostic test results following AFB microscopy, cytopathology, culture, RT-PCR (Multiplex), and GeneXpert (Figure 1). As we considered any positive test indicative of TB in line with clinical practice and used a composite reference standard, diagnostic yield was reported instead of test accuracy. We chose this approach due to the limitations of culture as a gold standard for LNTB, including its suboptimal sensitivity and a reduced incubation period of 8 weeks in our study.

### Sample size

We calculated the sample size considering the incidence of CL-TB in Bangladesh for the multi-country study. Assuming a CL-TB incidence of 3% among presumed participants presenting at a tertiary-level hospital derived from a working paper and a confidence interval (CI) of 95% with a precision rate of 3.5%, the sample size was estimated at 91 participants. We considered an attrition rate of 10%, bringing the final sample size to 101. However, we successfully enrolled 104 participants after screening 3,619 patients.

### Statistical analysis

We used Stata 15 (StataCorp LLC) for the statistical analysis of the data. Descriptive analysis determined the frequency and percentage of each variable. Chi-square tests were performed to assess the relationship between the independent and outcome variables. We used binary and multivariate logistic regression to estimate the association between outcome and independent variables following adjustments for statistically significant factors derived from the univariate analyses. Given the modest sample size (*N* = 104), we considered a *P* value of <0.10 and a 95% CI to indicate statistical significance during analysis, as this threshold has been recommended in small-sample studies to reduce the risk of Type II error and to help identify emerging patterns for further validation.

### Ethical statement

Since this was a multi-country study (PR-23031), the Ethical Review Committee of icddr,b, Leipzig University, Germany, and Makerere University, Uganda, approved it. We executed this study following the Declaration of Helsinki.

## RESULTS

### Diagnostic test results

Out of 52, cytopathology detected the highest number of CL-TB cases (*n* = 43), followed by GeneXpert (*n* = 37) and PCR (*n* = 35). In contrast, conventional diagnostic methods like AFB staining and culture showed considerably lower detection rates of 11.5% (*n* = 6) and 9.6% (*n* = 5), respectively. Only one participant was identified as a CL-TB case by all available diagnostic methods. Conversely, a non-TB mycobacterial infection was detected in one participant solely by RT-PCR (Multiplex), with all other diagnostic tests yielding negative results. The non-CL-TB participants were diagnosed as either metastatic carcinoma, neurofibroma, benign cysts, chronic non-specific lymphadenitis, Rosai–Dorfman disease, or lymphoproliferative disorder by cytopathology (see Supplementary Data on Figshare).

### Socio-demographic distribution of CL-TB

An overall review of the respondents’ socio-demographic information, including the distribution of CL-TB cases within the study population, is presented in a Supplementary Data in Figshare. The median age (IQR) of the participants was 30 years (IQR: 23–40). The distribution of age differed significantly between the CL-TB and non-CL-TB groups (*P* = 0.040). A higher proportion of CL-TB cases (63.5%) were within the 18–30 years age group compared to non-CL-TB participants (46.15%). Conversely, more non-CL-TB participants (28.85%) were aged above 45 years compared to CL-TB cases (9.6%). However, no significant differences were found between the two groups regarding sex distribution (male and female, both 46.15% and 53.85%, respectively, in each group), number of household members, education level, occupation, or family income.

### Other findings

The self-reported presence and duration of unexplained cough, low-grade fever, night sweats, and haemoptysis were similarly distributed between the CL-TB and non-CL-TB groups without significant differences (see Supplementary Data on Figshare).

Likewise, the distribution of smoking status, household or any other contact (e.g., hospital and workplace) with a TB patient, previous diagnosis of any form of TB, methods of previous TB diagnosis, and history of taking anti-TB medication was similar across the CL-TB and non-CL-TB groups, indicating no significant difference (see Supplementary Data on Figshare).

### Clinical characteristics

**Figure 2.**
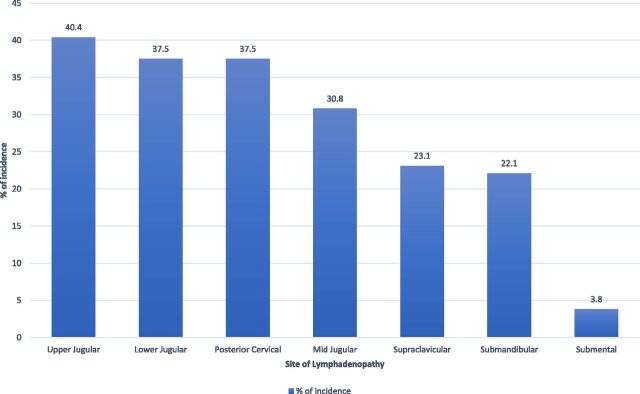
Distribution of the location of the affected cervical lymph nodes.

The mean body temperature of the participants at presentation was higher in the CL-TB group (36.5°C ± 0.11°C) compared to the non-CL-TB group (36.2°C ± 0.09°C). Fever (body temperature > 37.2°C) at presentation was observed more often among participants with CL-TB (21.2%) compared to the participants with non-TB cervical lymphadenitis (5.8%), and this difference was statistically significant (*P* = 0.041). Anaemia was clinically defined by lower palpebral subconjunctival pallor and was present in 53.8% of participants in both groups. However, no statistically significant differences were found between the groups in case of anaemia, body mass index distribution, participant-reported weight loss, and the presence of bacille Calmette-Guerin scar (see https://doi.org/10.6084/m9.figshare.30302923). Figure 2 depicts the distribution of affected LNs among participants. The upper jugular region shows the highest prevalence (40.4%), followed by the lower jugular and posterior cervical regions (both 37.5%). The remaining regions have lower frequencies, with the submental region having the least (3.8%). Half of the participants (51.9%) had LN swellings at multiple sites.

The median duration of LN enlargement was longer in participants with CL-TB (60 days, IQR: 23–180) compared to non-CL-TB participants (30 days, IQR: 15–120). However, LN size showed significant variation between the two groups (*P* = 0.02). The length of LNs greater than 1 cm was more common in the CL-TB group (86.5%) than in the non-CL-TB group (67.3%). Similarly, a width greater than 1 cm was observed in a higher proportion of CL-TB cases, with 75% of CL-TB participants compared to 59.6% in non-CL-TB individuals, and a significant difference in the distribution across width categories was observed (*P* = 0.012). However, the consistency (*P* = 1.000) and tenderness (*P* = 0.169) of the affected LNs did not differ significantly between the two groups (see https://doi.org/10.6084/m9.figshare.30302923).

The presence of a discharging sinus was noted in 9.6% of participants with CL-TB and in none among the non-CL-TB group, a finding that approached statistical significance (*P* = 0.057). Aspirate volume also tended to be significantly higher in CL-TB cases (*P* = 0.063). A significant difference was additionally noted in the appearance of aspirated materials (*P* < 0.001). While haemorrhagic aspirates predominated in both groups, they were more common among non-CL-TB participants (82.7%) than the comparison group (53.9%). In contrast, purulent aspirates were substantially more frequent in CL-TB cases (40.4%) compared to the non-CL-TB group (7.7%). The combination of a higher aspirate amount (>0.5 mL) and length (>1 cm) was significantly more common in CL-TB than in the comparison group (*P* = 0.019).

### Regression analysis

In the unadjusted model, participants in the younger age groups had higher odds of CL-TB compared to those aged over 45 years (*P* = 0.015). Several other clinical variables, such as body temperature, LN length, and aspirate amount, also demonstrated potential significance in univariate analysis (see https://doi.org/10.6084/m9.figshare.30302923). Notably, purulent aspirate emerged as an important associated factor (*P* = 0.015) for CL-TB diagnosis when adjusting for age, body temperature, LN length, and aspirate amount in our regression model. The youngest age of the participants showed significantly higher odds of CL-TB. In contrast, other clinical variables that showed potential significance in univariate analysis, such as body temperature, LN length, and aspirate amount, lost statistical significance in the multivariate model.

## DISCUSSION

CL-TB remains a prevalent form of EPTB in endemic regions, presenting ongoing challenges in early identification and management. In this study, we investigated the socio-demographic, clinical, and LN features associated with CL-TB in patients presenting at a tertiary care centre in Bangladesh. Furthermore, we assessed the case detection rates of the available diagnostic methods for CL-TB at the same centre. Understanding these factors is crucial for enhancing screening measures and improving diagnostic yield, ultimately leading to earlier case detection and treatment initiation. Our study revealed that CL-TB cases prevailed more in the younger age groups compared to individuals aged over 45 years in the unadjusted model. This aligns with the known epidemiological pattern of TB, which commonly affects economically active young adults in endemic areas,^10^ while the older age group (>45 years) suffers more from various co-morbidities that mimic TB symptoms, for example, malignancy.^11^

We observed no significant differences in sex distribution, indicating that CL-TB affects both sexes equally. However, several studies have reported contrasting findings.^12,13^ This discrepancy could potentially be attributed to the relatively smaller sample size of our study or variations in health care-seeking behaviours between genders in the specific population studied compared to others. Socio-economic factors such as household size, education level, and monthly family income did not significantly differ between the CL-TB and non-CL-TB groups in our cohort, probably due to the single-centre design and the specific demographic of patients accessing care at a tertiary referral centre, which may not fully represent the diverse socio-economic landscape of the entire country. The broader literature consistently highlights the strong link between poverty and EPTB, often mediated by factors like overcrowding and limited access to health care.^14^ To have a better understanding of the economic relationship with CL-TB, we need community-based studies.

In our study, clinical signs and LN characteristics provided valuable indicators for differentiating participants with CL-TB from non-TB cervical lymphadenitis with similar signs and symptoms. The presence of fever (>37.2°C) at presentation was significantly more frequent among cases in the unadjusted analysis. While this association was not statistically significant following adjustments, it remains a relevant clinical symptom, warranting consideration in presumptive CL-TB. Notably, CL-TB presented with significantly larger LNs, both in terms of length and width, compared to the non-CL-TB group. This finding is consistent with the pathological progression of TBLN, which typically involves significant lymphadenopathy.^15^ The appearance of the aspirate obtained from the LNs was a particularly strong differentiator. Purulent aspirate was significantly more common in CL-TB, while haemorrhagic aspirate was more prevalent in the comparison group. This reflects the caseating necrosis of the TB granulomas, leading to the formation of purulent material.^16^ The adjusted statistical analysis confirmed that haemorrhagic and other aspirate appearances were associated with lower odds of CL-TB compared to a purulent appearance, likely reflecting their non-specific nature and overlap with other pathological conditions such as malignancy or reactive lymphadenopathy.^17^ While a discharging sinus was observed only in cases and approached statistical significance, it was not a common feature in our cohort. These findings align with existing evidence that TB lymphadenitis typically presents as firm, matted nodes with caseous necrosis, distinguishing it from other lymphadenopathies.^18^

The rationale for investigating socio-demographic and clinical parameters stems from the diagnostic challenges associated with EPTB, where laboratory confirmation is often delayed or inaccessible. In our cohort, over 3,600 patients were screened, yielding a pre-test probability of approximately 1.5%. In such a context, identifying reliable clinical predictors, such as the youngest age and purulent aspirates, can help improve diagnostic triaging. These indicators could be used to augment pre-test probability, guide the appropriate selection of tests, and prioritise high-risk individuals for further workup. The identification of these socio-demographic and clinical factors associated with CL-TB has important implications for public health strategies in endemic regions. The finding that purulent appearance mediated several clinical associations, including age and fever, underscores its significance and supports its inclusion as a clinical indicator when evaluating presumptive cases of TBLN, particularly in high-burden settings. Integrating these clinical indicators into screening algorithms used by primary care providers could potentially improve the identification of individuals who require further diagnostic evaluation, contributing to minimised diagnostic delay, earlier detection, and improved patient management.

In our study, cytopathology detected most cases (82.7%) of CL-TB, outperforming molecular tools such as GeneXpert (71.2%) and PCR (67.3%). The superior diagnostic yield of cytopathology in this study reinforces its value as a preferred diagnostic method for TBLN.^4^ Consistent with this, findings from numerous studies conducted in other regions demonstrate the increased sensitivity of FNAC in diagnosing CL-TB.^18,19^ However, these findings contrast with studies conducted in developed countries with low TB prevalence, where the lower pre-test probability, lack of experience of the concerned pathologist, and unrepresentative sample of the studies may contribute to the significantly reduced sensitivity of FNAC (46%) in diagnosing TBLN.^20,21^ While FNAC cannot detect drug resistance patterns like molecular assays and culture,^4,22^ it has the added advantage of being able to suggest alternative diagnoses, such as reactive or malignant lymphadenopathy,^23^ thereby enhancing its overall diagnostic value in differentiating CL-TB from other causes of LN enlargement.

GeneXpert and PCR offered relatively high diagnostic yield in our study, making them indispensable for resource-constrained settings. These findings resembled other studies, demonstrating the high sensitivity of these molecular tests.^24,25^ While PCR showed a moderately high diagnostic yield in our study, it is important to contextualise its value compared to more integrated molecular platforms. RT-PCR, as employed here, can detect non-TB mycobacteria (NTM),^26^ which may explain some of the additional findings not picked up by other diagnostic methods. However, Xpert Ultra offers significant operational advantages – it targets IS6110 and IS1081 in addition to the rpoB gene, and its cartridge-based design enables simultaneous DNA extraction and amplification.^27^ Given these considerations, RT-PCR offers limited additional benefit over Xpert Ultra in routine diagnostic pathways. We acknowledge these limitations while interpreting PCR’s role in CL-TB diagnostics.

Microscopy for AFB, despite its utility, demonstrated significantly lower sensitivity (11.5%). The particularly low sensitivity of AFB microscopy in our study can be attributed to the paucibacillary nature of LN tissue in EPTB, where bacilli are often sparse and unevenly distributed, making microscopic detection difficult even in clinically apparent cases.^28^ Campelo et al.^29^ demonstrated similar findings. While capable of providing an initial indication of mycobacterial presence, this diagnostic procedure is subject to limitations imposed by low sensitivity, the extended time required for detection, the necessity of specialised technical skills, and the imperative of rapid specimen transit.^30^ This underscores the necessity of developing and implementing reliable, cost-effective, and alternative diagnostic methods, such as the lab-in-a-tube assay,^31^ especially in TB-endemic countries.

Despite being the gold-standard method for TB diagnosis,^4^ culture yielded low detection rates (9.6%) in identifying TB in the LN aspirates in our study. Several other studies showed similar findings.^29,30^ Multiple factors may contribute to this, including a low number of bacteria present in the small volume of aspirate obtained, the presence of bacteria sequestered within areas of caseous necrosis, and the possibility of reduced bacterial viability due to the invasive nature of the aspiration procedure.^28^ Despite the low sensitivity of TB culture and its extended turnaround time of over 8 weeks, culture remains essential for drug susceptibility testing (DST), especially in a setting like Bangladesh, where drug-resistant TB is a major concern.^4^ In this regard, extending the incubation period and using sterile and larger-volume samples of LN aspirate may improve diagnostic yield.^32^

We achieved the highest diagnostic yield for CL-TB in our study by utilising multiple diagnostic methods. The combination of cytopathology, GeneXpert, and culture detected 98.1% (*n* = 51) of cases, while the combination of cytopathology, culture, and PCR achieved a complete detection rate of 100% (*n* = 52). Similar results have been reflected in a previous study showing an increase in diagnostic accuracy by combining FNAC and PCR to diagnose TBLN,^33^ highlighting the synergistic roles of molecular, microbiological, and cytopathological diagnostics in accurately identifying CL-TB. This emphasises the importance of training initiatives on TB-PCR for the laboratory staff at tertiary-level hospitals. This strategy might leverage the existing RT-PCR laboratories used for COVID-19 and dengue and would decentralise diagnostic capacity while facilitating the establishment of TB-PCR services nationwide.

While this study provided valuable insights, it has limitations. The single-centre design may limit the representativeness of findings to other regions with differing TB burdens. To reduce the potential influence of this limitation, we selected a central medical facility that acts as a referral centre for the whole country. Additionally, the smaller sample size may have reduced generalisability. However, 3,619 patients were screened before enrolment, ensuring a robust selection process before enrolment and enhancing the reliability of the findings. Notably, a key strength of this study is that it is the first in Bangladesh to utilise LN aspirates directly for PCR testing, a method that is available but underutilised. In contrast to earlier studies that relied on PCR using the extracted DNA from culture growths,^34,35^ which can take up to 8 weeks with a low detection rate, the adoption of this direct aspirate method offers a practical solution to reduce diagnostic delays and may serve as a valuable model for future studies aiming to improve rapid TB diagnostics and therapy in resource-limited settings. The 8-week incubation period employed in our study resulted in a minimal number of positive cases (5/52) within the culture. Our findings revealed this critical gap in the national guideline, as its current recommendation of 2–8-week incubation period^4^ might be insufficient for paucibacillary tissue samples such as LN aspirates, which may require longer incubation, larger sample volumes, and avoidance of unnecessary decontamination of sterile samples to ensure accurate detection of *Mtb*.^32^

In this study, the combination of multiple diagnostic modalities demonstrated the highest case detection, emphasising the significance of a comprehensive diagnostic workup to detect CL-TB. Notably, this study is the first in Bangladesh to establish the utility of direct qPCR of LN aspirates for CL-TB diagnosis, facilitating timely treatment initiation, which is not possible with culture-based PCR. Nonetheless, culture and DST remain essential for cases unresponsive to initial therapy, especially in countries like Bangladesh, where drug-resistant cases are on the rise.^4^ Lastly, we recommend the development of a clinical algorithm that incorporates younger age and the presence of purulent aspirate as indicators for suspecting EPTB, alongside ensuring access to combined diagnostic approaches to facilitate timely and effective treatment and management of the disease.

## CONCLUSION

This study provides valuable epidemiological and clinical insights into CL-TB in a tertiary health care setting in Bangladesh. Younger age and purulent aspirate appearance emerged as strong, independent predictors of CL-TB. While cytopathology demonstrated the highest standalone diagnostic yield, combining it with molecular methods like GeneXpert or PCR achieved near-complete case detection. This integrated approach, however, complements rather than replaces the gold standard of culture, which remains essential for confirming viability and performing DST. Notably, another key innovation of this study was the demonstration of performing PCR directly from the LN aspirate, as opposed to the conventional practice of using cultured strains. Using this method, we suggested a rapid diagnostic solution that modifies the lengthy incubation period of traditional culture-based PCR methods. This approach is not only feasible but also offers a practical pathway to reduce diagnostic delays for CL-TB cases in an endemic setting like Bangladesh. Future multi-centre studies with larger cohorts are needed to validate these findings and support the integration of such strategies into national EPTB diagnostic guidelines.
